# Study on seismic vulnerability analysis of the interaction system between saturated soft soil and subway station structures

**DOI:** 10.1038/s41598-023-34658-y

**Published:** 2023-05-07

**Authors:** Xuelei Cheng, Qiqi Li, Ran Hai, Xianfeng He

**Affiliations:** 1 Yellow River Institute of Hydraulic Research, Zhengzhou, 450003 Henan China; 2 School of Architectural and Civil Engineering, Zhongyuan University of Technology, Zhengzhou, 450007 Henan China; 3 School of Traffic and Transportation Engineering, Dalian Maritime University, Dalian, 116026 Liaoning China

**Keywords:** Natural hazards, Solid Earth sciences, Mathematics and computing

## Abstract

The seismic vulnerability of interaction system of saturated soft soil and subway station structures was explored in this paper. The coupled nonlinear numerical models of interaction system were established using the *u*–*p* formulation of Biot′s theory to describe the saturated two-phase media. A refined finite element model of interaction system was developed to study its nonlinear seismic responses and seismic hazard mechanism. In this study, the multi-yield elastoplastic constitutive model was adopted for the soil, while a fiber section elastoplastic constitutive model was used for the structure. The seismic response of the structure was calculated by inputting the artificial seismic wave obtained from the power spectrum-triangular series method. The maximum inter-story drift angle was taken as a structural performance parameter for the subway station structure. The structural demand cloud was obtained under random ground motion sequences. Based on the probabilistic seismic demand model analysis method, the seismic vulnerability curve of the subway station structure was plotted, and the seismic vulnerability curve was analyzed as per the vulnerability of performance parameters. With the increase of soil strength, the vulnerability index of subway station structure under different peak acceleration ground motion decreased correspondingly. Based on the above vulnerability theory and analysis methods, it can be found from the above vulnerability theory and analysis methods that the subway station structure with established buried depth in saturated soft soil site exhibits a certain degree of safety and reliability, and can meet the seismic fortification goal of "no damage in small earthquakes, repairable in medium earthquakes and no collapse in large earthquakes". The results of vulnerability analysis are in line with the actual seismic survey, and the vulnerability analysis method proposed in this paper can be applied to the vulnerability analysis of underground structures on saturated soft soil foundation.

## Introduction

The seismic assessment of underground structures is one of the challenging issues in engineering design. This is because there are usually many sources of uncertainties in rocks and probable earthquake characteristics. It is therefore a new topic in the evaluation of the dynamic reliability of earthquake-damaged sturcutres^1^. Seismic vulnerability analysis was first applied the seismic performance studies of nuclear power plants in the 1970’s. While, with the continuous refinement and development of the performance-based probabilistic seismic vulnerability analysis method, it has gradually been applied to seismic studies of other infrastructures. As a method for evaluating the seismic performance of structures, seismic vulnerability analysis can determine the expected damage of structures or potential seismic hazards, and quantitatively evaluate the seismic performance of structures, that is, quantitatively describe the achievement of predetermined seismic performance targets of structures under different seismic fortification levels. It is of great significance to analyze the seismic vulnerability, predict their damage probability at all levels under different levels of earthquakes, and then evaluate their seismic performance and propose seismic reduction and isolation measures for the seismic design of underground structures. Although performance-based seismic design has been introduced into the current code for seismic design of underground structures in subways, its design and evaluation process is still underdeveloped and requires further research^2^.

Currently, seismic vulnerability analysis consists mainly of empirical method and theoretical analysis method. Due to the limitation of conditions, the empirical vulnerability curve is only applicable to the situation similar to the data source. The seismic response of underground structures varies under different seismic environments and site conditions, so the empirical vulnerability curves are difficult to generalize. The theoretical analysis method is a multiple calculation of the seismic response of underground structures, which is finally synthesized by regression. Commonly used calculation methods include response spectrum method, nonlinear static analysis method, and nonlinear time history analysis method.

Many scholars have conducted research toexplore the seismic vulnerability of structures. Torbol et al.^3^ investigated the effect of seismic wave incidence angle on the seismic vulnerability curve of bridge structure. Le et al.^4^ proposed a simple and comprehensive numerical analysis method based on the quasi-static analysis method and maximum likelihood estimation method considering SSI effect to analyze the seismic vulnerability of underground tunnels. He et al.^5^ defined and quantified five limit failure states of piers and bearings of isolated and non-isolated bridges on the basis of considering the randomness of bridge structure and ground motion parameters, and used a probabilistic demand analysis model to analyze the seismic vulnerability of seismically isolated and non-seismically isolated continuous girder bridges using the displacement ductility ratio of piers and columns and the relative displacement ratio of bearings as failure indicators, respectively. Tecchio et al.^6^ proposed a method for seismic vulnerability analysis of single span masonry arch bridge based on the limit analysis method. Liu et al.^7^ calculated the inter story displacement angle and tensile damage distribution of underground frame structure based on the dynamic incremental analysis method, defined the limit states through structural deformation and waterproof performance, and analyzed the seismic vulnerability of Dakai station in Japan. Argyroudis et al.^8^ proposed a numerical analysis method for seismic vulnerability suitable for shallow buried subway tunnel structures that considers the soil structure interaction (SSI) and aging effects due to corrosion of lining reinforcement. Liu et al.^9^ used the efficiency coefficient method to comprehensively evaluate the structural damage of the dam with displacement, stress and damage area as indicators, and applied the variable weight model to consider the influence of the index value on the index weight. Through calculation, the comprehensive damage index of the dam was obtained. According to the classification of dam seismic damage grades, the IDA method was improved by using the variable incremental step method, and the structural response process of the dam from the elastic state to complete damage under different intensity earthquakes was calculated and analyzed. Avanaki et al.^10^ investigated the effects of different composites of Steel FRC (SFRC), as the tunnel’s lining material, on its seismic vulnerability, compared to each other and to that of unreinforced and conventionally reinforced concrete cases, employing analytical fragility curves. Yigit et al.^11^ studied an area prone to earthquake-induced landslides in Istanbul to demonstrate the behavior of a natural gas pipeline network. It is located in the vicinity of the North Anatolian Fault Zone (NAFZ), where earthquakes of approximately 7.5 magnitude are expected to occur in Istanbul within the next few years. For this investigated region, the seismic vulnerability of natural gas pipelines subject to permanent ground deformation and seismic-wave propagation has been investigated, and risks have been highlighted. Using elastic beam theory, a new approximation has been developed to calculate earthquake. Moayedifar et al.^12^ used the incremental dynamic analysis (IDA) with 15 real earthquake records to evaluate the seismic response of a tunnel in south-west railway of Iran using different analytical methods. Based on a real underground structure called Daikai subway station in Japan, Xu et al.^13^ conducted an extended parametric study. In particular, a two-dimensional soil structure system was adopted for dynamic time-history analysis in that study. An equivalent linear model was adopted to consider the nonlinear behaviors of the soil elements, and an elastic model was used to simulate the structure elements. Huang et al.^2,14^ carried out a vulnerability assessment of circular tunnels in soft soil deposits in the Shanghai metropolitan subway system, accounting for the effects of soil-structure-interaction, local soil conditions and tunnel burial depth.

As can be easily seen, the existing structural seismic vulnerability analysis and findings have the following characteristics: (1) seismic vulnerability analysis is mainly applied to above-ground structures, such as bridge structures, building structures, hydraulic structures, and there are few reports on urban underground space structures; (2) due to the lack of seismic damage data of underground structures, scholars at home and aborad have conducted fewer studies on the seismic vulnerability of underground structures, but they mainly obtained seismic vulnerability curves through numerical simulations vulnerability curves. In addition, although the research on seismic vulnerability has achieved fruitful results, there are still some limitations in the field of engineering, for example: (1) In terms of seismic vulnerability expression, seismic vulnerability curves (functions) of structures are expressed in the form of limit state probability, which is not easy to be accepted by engineers; (2) In terms of the multi-level seismic vulnerability of a structure, the seismic vulnerability curve (function) of the structure is usually expressed as the probability of damage to the structure at multiple performance levels. Although this expression matches performance-based seismic needs, from the point of view of structural damage assessment, engineers prefer to use a single expression to evaluate the degree of damage to a structure rather than the probability of damage for multiple failure states.

In view of this, this paper establishes a fully-coupled effective stress refinement numerical solution model for the non-linear system of an underground station structure in a saturated soft soil site based on existing methods of structural seismic vulnerability analysis, using the underground station structure in a saturated soft soil site as the research context. And the seismic dynamic response of the buried structure is obtained by inputting the random ground motion into the established numerical model. The maximum inter story displacement angle was taken as the performance parameter of the subway station structure, and the cloud diagram relationship between the performance parameters of the underground structure and ground motion peak acceleration (PGA) was established. According to the probabilistic seismic demand analysis method, the seismic vulnerability curve of subway station structure was plotted and the vulnerability analysis based on performance parameters was carried out.

## Seismic vulnerability analysis method of underground structures

### Seismic vulnerability calculation process

In this paper, a specific analysis of the seismic vulnerability of an underground station structure on a saturated soft soil site is carried out in the following steps.

1. According to the site conditions of the underground building, 20 time histories of ground motion acceleration with the same dynamic characteristics suitable for soft soil sites were synthesized by simulating artificial seismic waves using the power spectrum triangular step method.
2. Based on the results of nonlinear dynamic incremental time history analysis (IDA, incremental dynamic analysis), the maximum inter story displacement angle was taken as the damage evaluation index of underground structure performance parameters, and the seismic vulnerability analysis was conducted based on the capacity demand model.
3. Through the regression analysis (least square method) of the response data under different ground motions, the cloud diagram relationship between the performance parameters of underground structures and the peak ground acceleration was established, and the structural demand response result function was obtained.
4. Based on the principle of vulnerability curve calculation, the vulnerability curves of underground structures under different ground motions were calculated, and the occurrence probability of structural failure state under the action of earthquakes of different hazard levels was compared and analyzed.
5. The concept of "vulnerability index" was introduced to evaluate the seismic damage of underground structures. The multi-level vulnerability curve based on probability expression was transformed into a non-probabilistic single parameter description based on vulnerability index.

**Figure 1:**
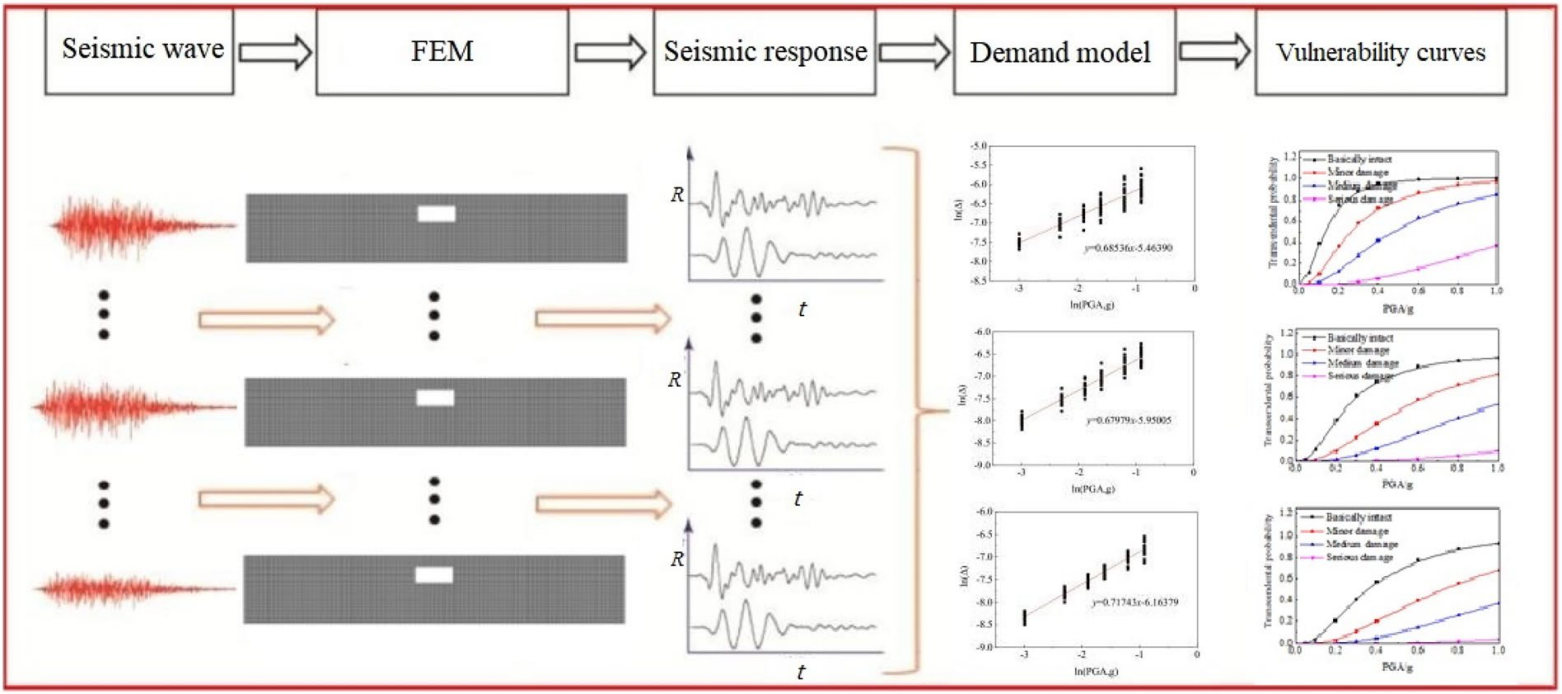
The flowchart of seismic vulnerability calculation of underground structure.

The calculation flow of seismic vulnerability of underground structures is shown in Fig. 1.

### Finite element calculation model

**Figure 2:**
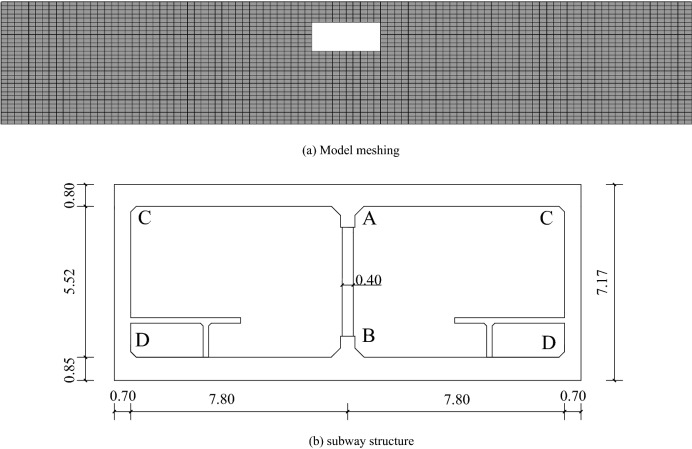
Finite element model of numerical calculation of structure under established buried depth conditions.

The numerical example is based on the typical single-story double-span subway station^15^, as shown in Fig. 2, in which the subway station structure buried depth is 5 m, its cross section size is: 17 m (width), 7.17 m (height), and 3.5 m (center column spacing). Figure 2 shows the finite element model mesh of the dynamic interaction system of the set subway station structure in soft soil interlayer site, the size of which is 170 m × 30 m. The site soil is simulated by quadUP soil and water coupling unit. The soil layer calculation parameters are combined with the experimental values and refer to the recommended values of OpenSEES clay constitutive elements.

In this paper, the saturated two-phase medium matrix numerical formula is^16^.

1 $$M\ddot{u} + C\dot{u} + \int_{\Omega } {B^{{\text{T}}} } \sigma^{^{\prime}} {\text{d}}\Omega - Qp = f^{{\text{u}}}$$

2 $$Q^{{\text{T}}} \dot{u} - S\dot{p} - H\dot{p} = f^{{\text{p}}}$$

where $M$ is the total mass matrix, $u$ is the displacement vector, $B$ is the strain–displacement matrix, $B \equiv LN^{u}$, which is related to the strain and displacement increment $d\varepsilon = Bd\overline{u}$; $\sigma^{\prime}$ is the effective stress tensor, $Q$ is the discrete gradient operator for soil–water coupling; $p$ is the pore pressure vector; $S$ is the compression coefficient matrix; $H$ is the permeability coefficient matrix. Vectors $f^{{\text{u}}}$ and $f^{{\text{p}}}$ respectively indicate the given boundary conditions of the volume force in the soil–water mixture and the liquid phase.

The multi-yield surface plastic constitutive equation is used for clay, and the yield surface formula of clay multi-yield surface model is

3 $$\begin{aligned} & f_{m} = \left\{ {\frac{3}{2}\left( {\tau - \alpha^{\left( m \right)} } \right):\left( {\tau - \alpha^{\left( m \right)} } \right)} \right\}^{\frac{1}{2}} - K^{\left( m \right)} = 0 \, \\ & \quad \quad \left( {m = 1,2, \cdots ,n} \right) \\ \end{aligned}$$

where $\tau$ is the partial stress tensor, *m* is the number of the *m*th yield surface, *m* ∈ (1, 2, …, *n*), *n* is the total quantity of yield surface. The parameters $\alpha^{\left( m \right)}$ and $K$ respectively indicate the center and radius of the *m*^*th*^ yield surface, $K^{\left( m \right)}$ is equal to $\sqrt {{3 \mathord{\left/ {\vphantom {3 2}} \right. \kern-0pt} 2}}$ times of the radius of the yield surface. The double dot product of tensor $A$ and $B$ is $A:B = A_{ij} :B_{ij}$.

The plastic constitution of clay multi-yield surface adopts the law of partial kinematic hardening, and the movement direction tensor of yield surface is defined as

4 $$\mu = \left[ {s_{{\text{T}}} - \alpha^{m} } \right] - \frac{{M_{m} }}{{M_{m + 1} }}\left[ {s_{{\text{T}}} - \alpha^{m + 1} } \right]$$

where $s_{{\text{T}}}$ is the second-order deviatoric stress tensor, which represents the deviatoric stress tensor of the intersection point of yield surface *f*_*m *+ 1_ and *f*_*m*_; $\alpha_{m}$ and $\left( {p^{\prime} + p^{\prime}_{0} } \right)\alpha_{m + 1}$ are the center of yield surface *f*_*m*_ and *f*_*m *+ 1_ respectively.

**Figure 3:**
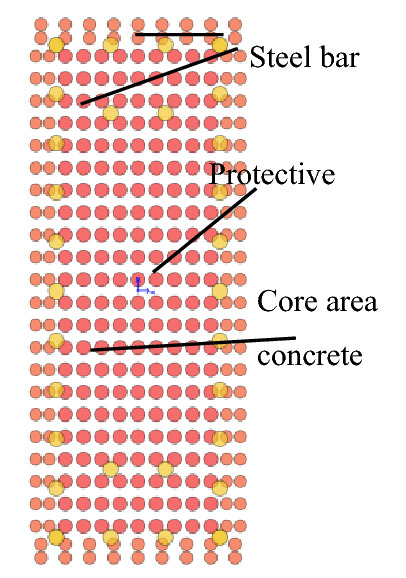
Schematics of fiber cross section.

**Figure 4:**
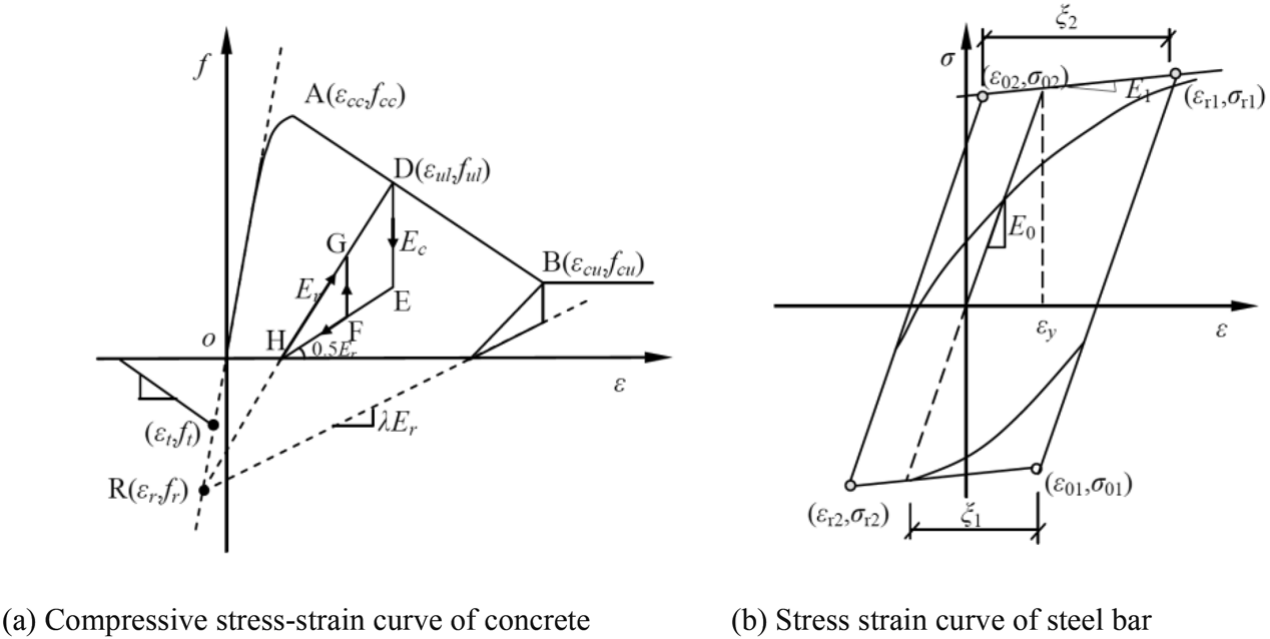
The constitutive model of reinforced concrete.

The designed strength of the concrete with established embedded depth is C40 and the density is 2500 kg/m^3^. The adoption of fiber section unit of the underground structure considers its nonlinear dynamic performance. The schematic diagram of the fiber section is shown in Fig. 3. Specifically, the Concrete02 constitutive model (modified Kent-Park concrete model) is used in the structural concrete, and the stress–strain relationship is shown in Fig. 4a. The steel bar uses Steel02 dynamic isotropic hardening bilinear material model, and the stress–strain relation is shown in Fig. 4b, in which the elastic modulus of steel bar is 200 GPa, and the yield strength is 400 MPa.

In this paper, the calculations involve 3 different site types (soft soil site, medium soft site and hard soil site). The values of the calculated parameters for the soil composition are presented in Table 1.

**Table 1:** Model parameters of middle soft clay.

Parameters	Density *ρ* (kg m^−3^)	refShearModul *G*_*ref*_ (MPa)	refBulkMoudl *B*_*ref*_ (MPa)	Cohesi *c* (kPa)	PeakShearStra *γ*_max_	Permeability coefficient *k* (m s^−1^)	Porosity *n*
Soft clay	1700	17.0	79	18	0.1	3.0 × 10^−7^	0.6
Medium soft site	1880	75.2	351	37	0.1	3.0 × 10^−7^	0.5
Stiff soil site	2000	180.0	840	75	0.1	3.0 × 10^−7^	0.4

Density is saturated soil mass density;

refShearModul is reference low-strain shear modulus, specified at a reference mean effective confining pressure refPress;

refBulkMoudl is reference bulk modulus, specified at a reference mean effective confining pressure refPress;

Cohesi is apparent cohesion at zero effective confinement;

PeakShearStra is an octahedral shear strain at which the maximum shear strength is reached, specified at a reference mean effective confining pressure refPress.

### Artificial seismic wave

The input of ground motions has a direct impact on the seismic performance of underground structures. For the seismic vulnerability analysis of underground structures, it is essential to select ground motions that are appropriate to the characteristics of the site. Typically, there are two types of ground motions used in calculations, one is the selection of appropriate grounds motion from the database, and the other is synthetic ground motions. Due to the limitation of the number of actual ground motion samples, the number of natural seismic waves that meet the actual engineering site conditions is relatively small and can hardly meet the requirements of actual design and analysis. Therefore, using synthetic ground motion is a common method used in vulnerability analysis. In this way, artificial seismic waves meeting the corresponding requirements need to be generated for structural dynamic time course analysis based on the site conditions, seismic intensity, and other information.

**Figure 5:**
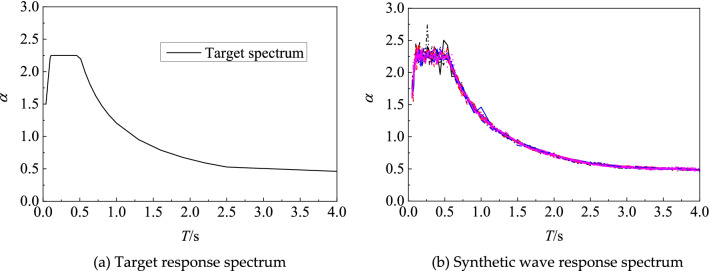
The response spectrums of input ground motion acceleration.

According to the engineering site type, the design response spectrum in the code for seismic design of buildings (GB 5011-2010) is applied as the target spectrum, and based on the power spectrum trigonometric series method, 20 artificial seismic waves suitable for soft soil site were generated from the initial conditions such as characteristic period, maximum value of horizontal seismic wave influence coefficient and seismic wave amplitude to provide seismic wave sources for the underground structural dynamic time course analysis method. The standard design response spectrum had an Eigen period of 0.55 s, a platform amplification factor of 2.25, and an overall scaling factor of 1.0. The target response spectrum was determined according to the specification for artificial wave making, followed by response spectrum fitting and baseline correction^17^. The error between the artificially generated seismic wave acceleration response spectrum curve and the designed seismic acceleration response spectrum curve was less than 5%. As shown in Fig. 5, the target spectrum of ground motion acceleration and artificial synthetic wave response spectrum were input. The uncertainty of ground motion was reflected by the discreteness of changes for different ground motion time history curves. As can be seen from the figure, the ground motion accelerations of 20 bars were quite different and discrete, but the predominant periods were basically the same. The peak acceleration (PGA) of the generated artificial seismic wave was modulated proportionally. Table 2 lists the corresponding relationship between seismic fortification intensity and design basic seismic acceleration. Six ground motion peak accelerations of 0.05 g, 0.10 g, 0.15 g, 0.20 g, 0.30 g and 0.40 g have been selected for analysis in the range of 0.1 to 1.0 g.

**Table 2:** The relationship between the earthquake intensity and the peak acceleration.

Seismic fortification intensity	VI	VII	VIII	IX
Design basic seismic acceleration value	0.05 g	0.10 (0.15)g	0.20 (0.30)g	0.40 g

The principle of generating artificial seismic wave by the power spectrum trigonometric series method is as follows:

Non-stationary seismic acceleration is regarded as the product of a steady-state stochastic process and an envelope function that takes into account non-stationary characteristics:

5 $$a\left( t \right) = f\left( t \right)a_{s} \left( t \right)$$

where *a*(*t*) is the time history of seismic acceleration, *f*(*t*) denotes the envelope function and *a*_s_(*t*) represents a Gaussian stationary random process with zero mean and (unilateral) power spectral density function. The function expression of *f*(*t*) can be seen as follows:

6 $$f\left( t \right) = \left\{ {\begin{array}{*{20}l} {\left( {{t \mathord{\left/ {\vphantom {t {t_{1} }}} \right. \kern-0pt} {t_{1} }}} \right)^{2} } \hfill & {\quad t < t_{1} } \hfill \\ 1 \hfill & {\quad t_{1} \le t \le t_{2} } \hfill \\ {{\text{e}}^{{ - c\left( {t - t_{2} } \right)}} } \hfill & {\quad t_{2} \le t \le t_{3} } \hfill \\ {0} \hfill & {\quad t_{3} \le t \le T} \hfill \\ \end{array} } \right.$$

where *t*_1_, *t*_2_, *t*_3_ and *T* are the start and end times of seismic wave stabilization period, the end time of attenuation period and the total duration of seismic wave, respectively and *c* denotes the constant controlling the decay rate.

For the Gaussian stochastic random process represented by *a*_s_(*t*), the triangular series cosine function model is adopted for synthesis and the specific expression is:

7 $$a_{s} \left( t \right) = \sum\limits_{k = 1}^{N} {C_{k} } \cos \left( {\omega_{k} t + \varphi_{k} } \right)$$

where *φ*_*k*_ is the phase angle randomly and uniformly distributed in (0, 2π), *ω*_*k*_, and *C*_*k*_ are represent the frequency and amplitude of the kth spectral component respectively and *ω*_*k*_ and *C*_*k*_ are determined according to the given power spectral density function.

8 $$\left\{ \begin{gathered} C_{k} = \sqrt {4S\left( {\omega_{k} } \right)\Delta \omega } \hfill \\ \Delta \omega = {{2{\uppi }} \mathord{\left/ {\vphantom {{2{\uppi }} T}} \right. \kern-0pt} T} \hfill \\ \omega_{k} = k\Delta \omega \hfill \\ \end{gathered} \right.$$

where *S*(*ω*_*k*_) is a given power spectral density function.

The relationship between standard acceleration response spectrum and power spectral density function is given below:

9 $$S_{x} \left( \omega \right) = {{\frac{\xi }{{{\uppi }\omega }}\left[ {S_{a}^{T} \left( \omega \right)} \right]^{2} } \mathord{\left/ {\vphantom {{\frac{\xi }{{{\uppi }\omega }}\left[ {S_{a}^{T} \left( \omega \right)} \right]^{2} } {\ln \left[ {\frac{{ - {\uppi }}}{\omega T}\ln \left( {1 - P} \right)} \right]}}} \right. \kern-0pt} {\ln \left[ {\frac{{ - {\uppi }}}{\omega T}\ln \left( {1 - P} \right)} \right]}}$$

where $S_{a}^{T} \left( \omega \right)$ represents the given target acceleration response spectrum, *ξ* denotes the given damping ratio and *P* is the response transcendental probability, *P* ≤ 15%.

### Definition of structural damage state and performance level

As per the code for seismic design of buildings (GB 5011-2010), the damage state of the structure is divided into three fortification levels of "no damage in small earthquake, repairable in medium earthquake and no collapse in large earthquake". According to the code for seismic design of urban rail transit structures (GB 50909-2014), the seismic performance requirements of urban rail transit structures are also divided into three levels. During the seismic vulnerability analysis of underground structures, the definition of structural damage state can greatly affect the shape of vulnerability curve. To reflect different levels of structural performance in practical engineering design, it is necessary to provide a quantitative description of the performance levels, which are related to the damage state of the structures. Among other things, the failure state of the structure can be determined by its seismic response parameters or damage indexes. Therefore, these parameters can be used to quantitatively describe different levels of structural performance. Previous studies have shown that the seismic performance level of frame structure largely depends on the structural deformation (such as inter story displacement angle, vertex displacement, plastic hinge angle, displacement ductility coefficient and other structural response parameters), that is, the structural deformation can reflect its overall performance^18^. Among the structural deformation parameters, the floor displacement angle was selected in this paper as its calculation method of the floor displacement angle is relatively simple and is consistent with the performance index in the current design codes in China.

**Figure 6:**
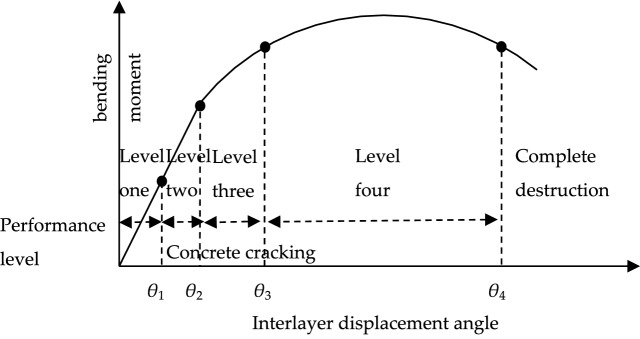
Structural performance curve.

The numerical example adopted the performance level and quantitative index of subway underground structure. The quantitative index was mainly based on the results of numerical simulation analysis of typical underground structure. Meanwhile, the limit values of the inter story displacement angles corresponding to the four performance levels of rectangular subway underground structure were analyzed with reference to the test statistics and the research requirements of current codes. As shown in Table 3, the seismic performance level of subway underground structures was divided, and the corresponding quantitative indexes of each structural performance state can be seen in Fig. 6.

**Table 3:** Performances level and quantitative indicators of subway station structure.

Performance level	Structural state	Quantitative index	Performance description
Level one	Basically intact	*θ*_1_ = 1/1000	The construction member is partially cracked and can be used without repair, and the structure is in the elastic stage
Level two	Minor damage	*θ*_2_ = 1/600	Slight damage, with individual frame columns yielding in the tensile reinforcement and the structure entering the elastic stage
Level three	Medium damage	*θ*_3_ = 1/400	The unconstrained concrete of some frame columns peeled off, which can be restored to use after emergency repair, and some parts of the structure entered the elastic–plastic stage
Level four	Serious damage	*θ*_4_ = 1/200	The main frame columns enter the limit state of bearing capacity, the damage is serious but does not collapse, and the structure enters the elastic–plastic stage

### Principle of seismic vulnerability analysis based on probabilistic seismic demand model

The purpose of probabilistic seismic demand model is to establish a probabilistic relationship between structural demand and seismic peak acceleration through regression analysis. The probability that an underground structure reaches or exceeds a certain limit failure state (substantially intact, slightly damaged, moderately damaged and severely damaged) is as follows:

10 $$P\left( {{\text{LS}}} \right) = P\left[ {D \ge C|IM} \right]$$

where *P*(LS) is the probability that the structure reaches and exceeds a certain limit state under the action of ground motion, *IM* refers to the Intensity Measure, such as PGA, *S*_a_(*T*_1_, 5%), *C* is the seismic capacity of the structure, and *D* denotes the seismic demand capacity that is the structural demand under different levels of earthquake.

Given that the structural seismic demand *D* follows a log-normal distribution, the relationship between the median structural seismic demand $$\overset{\lower0.5em\hbox{$\smash{\scriptscriptstyle\frown}$}}{D}$$ and the peak ground acceleration PGA obeys an exponential relationship as follows:

11 $$\overset{\lower0.5em\hbox{$\smash{\scriptscriptstyle\frown}$}}{D} = a\left( {PGA} \right)^{b}$$

Logarithm of Eq. (11)

12 $$\ln \overset{\lower0.5em\hbox{$\smash{\scriptscriptstyle\frown}$}}{D} = \ln a + b\ln \left( {PGA} \right)$$

where *a*, *b* are the exponential relationship parameters, which can be obtained by fitting the data after incremental dynamic analysis. If the logarithmic form of structural seismic demand $\ln D$ satisfies the normal distribution, the logarithmic median $\lambda_{d}$ and logarithmic standard deviation $\beta_{d}$ of structural seismic demand parameters at this time are as follows respectively

13 $$\lambda_{d} = \ln \overset{\lower0.5em\hbox{$\smash{\scriptscriptstyle\frown}$}}{D}$$

14 $$\beta_{d} = \sqrt {\frac{1}{N - 2}\sum\limits_{i = 1}^{N} {\left( {\ln \left( {d_{i} } \right) - \ln \overset{\lower0.5em\hbox{$\smash{\scriptscriptstyle\frown}$}}{D} } \right)^{2} } }$$

where *N* is the number of ground motions for the nonlinear time history analysis and *d*_*i*_ represents the *i*th peak ground motion demand.

The probability of structural failure under earthquake can be rewritten as:

15 $$P\left( {{\text{LS}}} \right) = P\left[ {C/D < 1} \right]$$

or

16 $$P\left( {{\text{LS}}} \right) = P\left[ {C - D < 0} \right]$$

However, in the study of seismic vulnerability, the structural capacity function and seismic demand function obey lognormal distribution, specifically, order *Z* = ln*C *− ln*D*, then $\lambda_{z} = \lambda_{c} - \lambda_{d}$, $\beta_{z} = \sqrt {\beta_{c}^{2} + \beta_{d}^{2} }$, where, $\lambda_{c}$ is the logarithmic median of structural seismic capacity, and $\beta_{c}$ represents the logarithmic standard deviation of structural seismic capacity.

Therefore, the failure probability of the structure under earthquake can be expressed as:

17 $$P\left( {{\text{LS}}} \right) = P\left( {Z < 0} \right) = \int_{ - \infty }^{0} {\frac{1}{{\beta_{z} \sqrt {2{\uppi }} }}} {\text{e}}^{{\left[ { - \frac{1}{2}\left( {\frac{{Z - \lambda_{z} }}{{\beta_{z} }}} \right)^{2} } \right]}} {\text{d}}Z$$

Convert Eq. (17) to standard normal distribution, order ${\text{d}}Z = \beta_{z} {\text{d}}t$, $Z = \lambda_{z} + t\beta_{z} < 0$, then, $t < \frac{{\lambda_{z} }}{{\beta_{z} }}$.

Vulnerability model Eq. (17) can be rewritten as

18 $$\begin{aligned} P\left( {{\text{LS}}} \right) & = P\left( {t < - \frac{{\lambda_{z} }}{{\beta_{z} }}} \right) = \int_{ - \infty }^{{ - \frac{{\lambda_{z} }}{{\beta_{z} }}}} {\frac{1}{{\sqrt {2{\uppi }} }}} {\text{e}}^{{\left[ { - \frac{{t^{2} }}{2}} \right]}} {\text{d}}Z = \Phi \left( { - \frac{{\lambda_{z} }}{{\beta_{z} }}} \right) \\ & = \Phi \left( { - \frac{{\lambda_{c} - \lambda_{d} }}{{\sqrt {\beta_{c}^{2} + \beta_{d}^{2} } }}} \right) = \Phi \left( { - \frac{{\ln \overset{\lower0.5em\hbox{$\smash{\scriptscriptstyle\frown}$}}{C} - \ln \overset{\lower0.5em\hbox{$\smash{\scriptscriptstyle\frown}$}}{D} }}{{\sqrt {\beta_{c}^{2} + \beta_{d}^{2} } }}} \right) \\ \end{aligned}$$

where $$\overset{\lower0.5em\hbox{$\smash{\scriptscriptstyle\frown}$}}{C}$$ is the structural bearing capacity corresponding to a certain limit failure state, generally taking the median of failure index.

Substitute Eq. (12) into Eq. (18)

19 $$P\left( {{\text{LS}}} \right) = \Phi \left( {\frac{{\ln \left( {{{\overset{\lower0.5em\hbox{$\smash{\scriptscriptstyle\frown}$}}{D} } \mathord{\left/ {\vphantom {{\overset{\lower0.5em\hbox{$\smash{\scriptscriptstyle\frown}$}}{D} } {\overset{\lower0.5em\hbox{$\smash{\scriptscriptstyle\frown}$}}{C} }}} \right. \kern-0pt} {\overset{\lower0.5em\hbox{$\smash{\scriptscriptstyle\frown}$}}{C} }}} \right)}}{{\sqrt {\beta_{c}^{2} + \beta_{d}^{2} } }}} \right) = \Phi \left( {\frac{{\ln \left[ {\frac{{{\text{e}}^{a} \left( {PGA} \right)^{b} }}{{\overset{\lower0.5em\hbox{$\smash{\scriptscriptstyle\frown}$}}{C} }}} \right]}}{{\sqrt {\beta_{c}^{2} + \beta_{d}^{2} } }}} \right)$$

When PGA is used as the ground motion parameter, $\sqrt {\beta_{c}^{2} + \beta_{d}^{2} }$ is taken as 0.5; When *S*_a_ is used as the ground motion parameter, $\sqrt {\beta_{c}^{2} + \beta_{d}^{2} }$ is taken as 0.3.

According to Eq. (19), the transcendental probability of each limit failure state of the underground structure can be obtained, and then the failure probability *P*(DS) of each state of the structure under different intensity earthquakes can be evaluated according to the seismic vulnerability curve, so as to further guide the seismic design of the structure.

**Figure 7:**
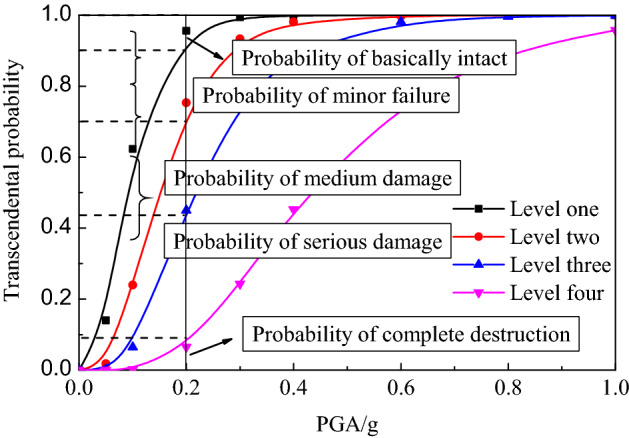
Damage state probability curves of underground structure.

The occurrence probability *P*(DS) of structural failure state is expressed as the difference of the transcendental probability of adjacent states:

20 $$P\left( {{\text{DS}}_{j} } \right) = \left\{ {\begin{array}{*{20}l} {1 - P\left( {{\text{LS}}_{{1}} } \right),} \hfill & {\quad j = 1} \hfill \\ {P\left( {{\text{LS}}_{j - 1} } \right) - P\left( {{\text{LS}}_{j} } \right),} \hfill & {\quad j = 2,3, \ldots ,N} \hfill \\ {P\left( {{\text{LS}}_{N} } \right),} \hfill & {\quad j = N + 1} \hfill \\ \end{array} } \right.$$

where *N* is the number of ultimate failure states. According to the relationship between limit state and failure state, the underground structure is divided into *N* + 1 failure state under *N* limit state. In this paper, four limit states of basically intact, slight damage, medium damage and serious damage were adopted. These classify the structure into five failure states: basically intact (DS_1_), slight damage (DS_2_), medium damage (DS_3_), serious damage (DS_4_) and complete damage (DS_5_), as shown in Fig. 7.

A total of 20 synthetic seismic waves, 6 seismic peak acceleration levels and 13 operating conditions were calculated in this paper. Each calculation took about 1 h, and the total calculation time required took 1560 h.

## Seismic vulnerability analysis of subway station structure under different site conditions

**Figure 8:**
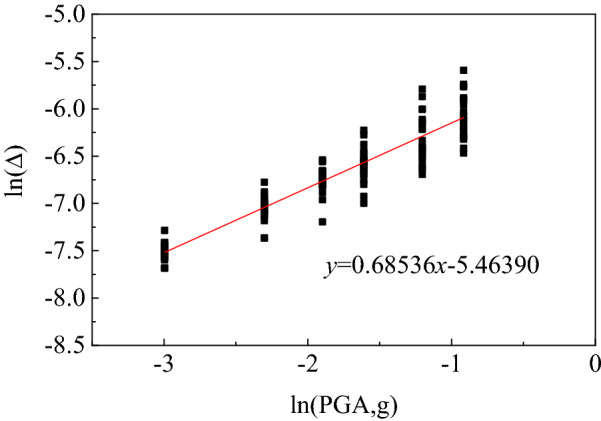
Regressive analysis of damage index of underground structure in soft soil.

As shown in Fig. 8, the results of logarithmic regression analysis between the maximum inter story displacement angle response value of the subway station structure and the independent variable peak acceleration PGA under different site types were obtained, where the abscissa represented the logarithm of the peak acceleration of ground motion, and the ordinate represented the logarithm of the maximum performance parameters of subway underground structure under the action of earthquake of that intensity. By adjusting the parameter regression analysis ln(Δ) and ln(PGA), it could be found that the relevant parameters obtained from the linear regression exhibited a high correlation, so the univariate linear regression method *y* = *a* + *bx was* applied to establish the logarithmic linear relationship between them. The structural demand response structural function is listed in Table 4. According to the goodness of fit, the method shows a good fitting performance.

**Table 4:** Demand response function of subway station structure in different site condition.

Site condition	Fitting function	*R*-squared
Soft clay	$$\ln \overset{\lower0.5em\hbox{$\smash{\scriptscriptstyle\frown}$}}{D} = 0.68536\ln \left( {PGA} \right) - 5.4639$$	0.86604

**Figure 9:**
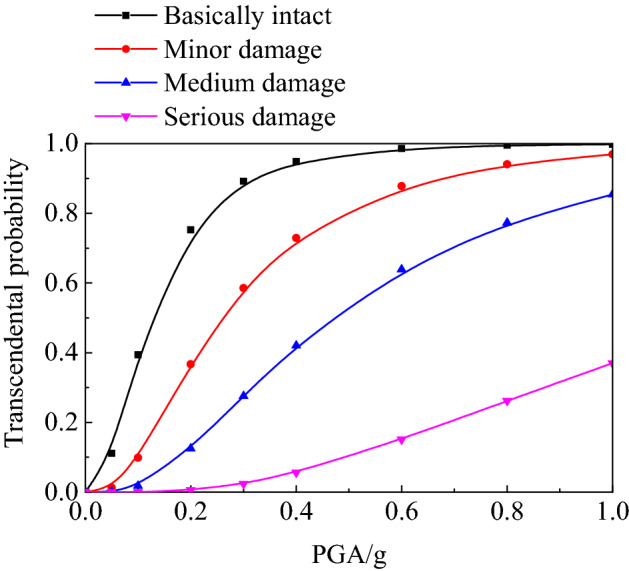
Seismic vulnerability curves of underground structure in soft soil.

A MATLAB program was prepared using the principle of vulnerability curve calculation to calculate the vulnerability curve of subway station structure under different input acceleration peak ground motion under different site types, as shown in Fig. 9. It can be seen from the figure that with the increase of seismic peak acceleration, the slope of vulnerability curve first increased and then decreased, the performance level of the structure gradually developed from basically intact to severely damaged, the transcendental probability of damage at each structural level increased in varying degrees, and the structural vulnerability curve tended to be flat with the severity of structural damage. In particular, the probability of exceedance increased rapidly for substantially intact and slightly damaged, while it increased slowly for moderately damaged and severely damaged.

It can be seen from Fig. 6 that when PGA is less than 0.1 g (with the seismic intensity bejing VII moderate earthquake and below), the transcendental probability of a basic intact state of the underground structure is less than 30%, and the basic intact seismic performance standard can be ensured. Within the range of 0.1–0.2 g (with the seismic intensity iranging from VII to VIII), the transcendental probability limit of the basic intact state of the underground station structure is 70%, and the basic intact seismic performance standard can no longer be guaranteed. When PGA reaches 0.3 g, the transcendental probability of slight damage of the structure tends to be 1.0, which means that it is basically impossible for the structure to remain in good condition. In the strong earthquake range of 0.2–0.4 g (with the seismic intensity iranging from VIII to IV), the subway station structure is mainly slightly damaged. Specifically, 70% of the transcendental probability reaches a state of slight damage and 40% of the transcendental probability reaches a state of moderate damage. When PGA is 0.4 g or higher (seismic intensity is above IX), the transcendental probability of serious damage to the structure increases significantly, with the probability of exceeding the limit reaching over 50%.

**Figure 10:**
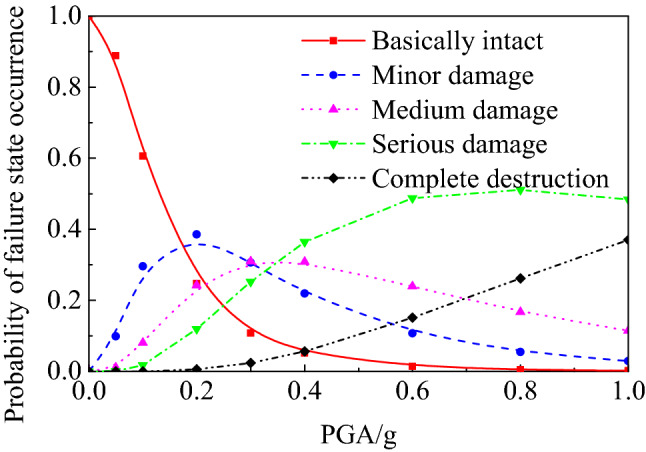
Damage state probability curves of subway station structure in soft soil.

A comparative analysis of the damage probability curves of the subway station structures was carried out based on the results of the calculation of limit state failure probability, and the results are shown in Fig. 10. It can be seen from the figure that the failure state probability curve of subway station structure does not monotonically increase with the increase of ground motion peak acceleration as the seismic vulnerability curve (limit state probability curve), but there is a falling section. This indicates that the failure state of underground structures is constantly changing under the action of ground motion with different peak accelerations.

**Figure 11:**
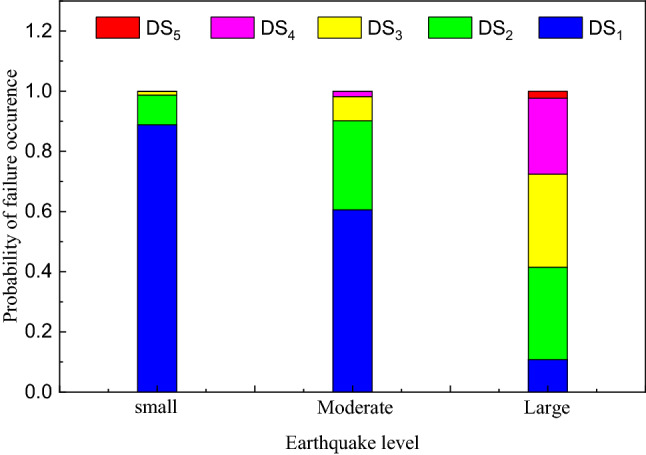
Damage state probabilities of the structure under the earthquakes with different hazard levels.in different cases of site condition.

According to the code for seismic design of building structures (GB 50011-2010), the peak ground acceleration corresponding to small earthquake, medium earthquake and large earthquake for the subway station structure in this example are 0.05 g, 0.13 g and 0.28 g, respectively. Figure 11 shows the failure probability of subway station structure in failure state under different site conditions when the ground motion intensity reaches the level of minor earthquake, medium earthquake and large earthquake. It can be seen from the figure that, according to the three-level requirements of seismic design, when the local vibration intensity reaches the minor earthquake level, the probability of subway station structure in basically intact failure state is much greater than that the other failure states. It can be considered that the structure is basically controlled below the minor failure level at this time, satisfying the seismic fortification goal of "minor earthquake is not bad"; when the local vibration intensity reaches the medium earthquake level, the probability that the subway station structure is basically intact decreases, and the failure probability of minor damage state increases significantly. At the same time, the probability of failure in the moderate damage state of the structure increases slightly. However, the sum of the probability of the structure being in these two states was significantly greater than the probability of failure in other failure states, satisfying the seismic fortification target of "medium earthquake repairable"; when the local vibration intensity reaches the large earthquake level, the failure state probability of the subway station structure corresponding to medium failure, serious failure and serious failure increases significantly, indicating that the degree of structural damage gradually deepens, but the overall failure level of the subway station structure still met the seismic fortification goal of "not falling in a large earthquake". In addition, comparing the probability of failure of subway station structures in different site categories under different hazard levels of ground motion, the level of structural failure for soft soil sites is significantly greater than for medium to soft and hard soil sites.

## Vulnerability index

Given that the probability of occurrence of failure state is not easily accepted by engineering personnel, the damage index of underground structures is defined by the seismic damage index adopted in China's post-earthquake investigation. The seismic susceptibility results were applied to calculate the probability of failure for different failure states of underground structures, and the mathematical expectation of seismic damage index was used as vulnerability index (VI, vulnerability index) to evaluate the seismic safety of underground structures. As an extension of the traditional seismic vulnerability analysis, the seismic vulnerability index transforms the multi-level vulnerability curve based on probability expression into a non-probabilistic single parameter description based on vulnerability index, which will be beneficial for the wide application of seismic vulnerability analysis structures in engineering practice.

The vulnerability index can be defined as follows:

21 $$FI = \sum\limits_{j = 1}^{n} {DF_{j} } \times P\left( {DS_{j} |PGA} \right)$$

where *n* is the number of failure states of underground structures, $P\left( {DS_{j} |PGA} \right)$ is the occurrence probability corresponding to the *j*th failure state under the action of peak acceleration ground motion and $DF_{j}$ is the seismic damage index corresponding to the failure state, as listed in Table 5.

**Table 5:** Damage states and the corresponding damage factor.

Damage index	Damage state				
	Basically intact	Minor damage	Medium damage	Serious damage	Complete destruction
Upper and lower limits/(%)	[0,10]	[10,30]	[30,55]	[55,85]	[85,100]
Average value/(%)	5	20	42.5	70	92.5

**Figure 12:**
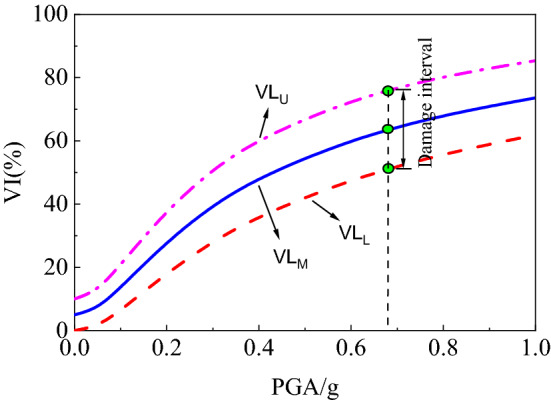
Seismic vulnerability factor curves.

The vulnerability index is expressed as a parameter between 0 and 1. The mathematical expectation of the seismic damage index of the single structure can be calculated in combination with the failure probability of the structural failure state obtained based on the vulnerability analysis to quantitatively evaluate the seismic damage of the structure. DF_*j*,L_, DF_*j*,U_ and DF_*j*,M_ are respectively used to represent the lower limit, upper limit and average value of seismic damage index DF_*j*_ (as listed in Table 5), which are substituted into Eq. (21) to obtain the vulnerability index curve of the subway station structure, as shown in Fig. 12. In the figure, VIL, VIU and VIM represent the lower limit, upper limit and average value of structural vulnerability index of subway station respectively. The vulnerability index interval corresponding to a particular peak seismic acceleration PGA can be calculated on the damage curve, and by comparing it with the empirical values of the damage index in Table 5, the degree of damage to the structure can be evaluated quantitatively.

**Figure 13:**
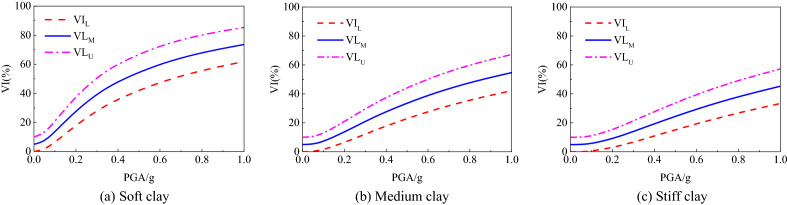
Vulnerability index of subway station structure in different cases of site condition.

**Figure 14:**
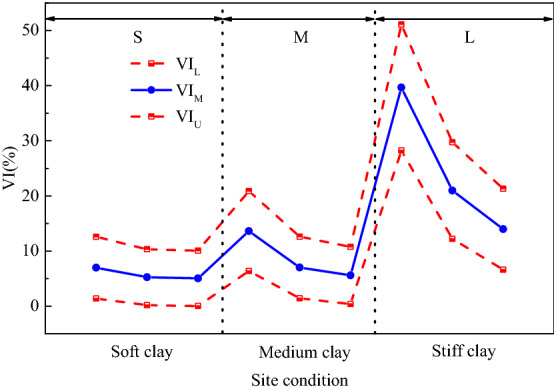
Vulnerability index conditioned on FE, DBE and MCE intensities for underground structure in different cases of site condition.

The upper and lower limits and average values of the failure probabilities obtained in Fig. 12 and the seismic damage index in Table 5 were substituted into Eq. (21) to calculate the vulnerability index of subway station structure under different site types and conditions under earthquake, as shown in Fig. 13. In order to further comprehensively evaluate the seismic performance of subway station structure, the vulnerability index interval under small, medium and large earthquakes specified in code for seismic design of buildings (GB 50011-2010) was calculated, as shown in Fig. 14. It can be seen that the vulnerability index of subway station structure in soft soil site under small earthquake is not more than 20%, the vulnerability index under medium earthquake is not more than 30%, and the vulnerability index under large earthquake is not more than 50%. Compared with the empirical earthquake damage index in Table 5, it can be seen clearly that under the action of small and medium earthquakes, the seismic damage of subway station structure did not exceed the slight damage level; under the action of large earthquake, the seismic damage of subway station structure was basically controlled at the medium damage level, and no more serious damage occurred. This shows that the reinforced concrete subway station structure studied in this paper fulfils the seismic performance requirements of "no damage in small earthquake", "repairable in medium earthquake" and "no collapse in large earthquake" in China's seismic code. With the increase of soil strength, the vulnerability index of subway station structure under different peak acceleration ground motion decreased correspondingly. This suggests that the occurrence of severe damage can be better controlled for subway station structures on medium to soft sites and hard soil sites under the action of large earthquakes.

## Conclusion

This paper combines the current status and characteristics of existing results on the seismic vulnerability of underground structures, and takes the seismic performance of underground structures in saturated soft soil sites as the research background. Stochastic ground motion is put into the numerical model of the established buried structure, and the maximum inter story displacement angle is used as the performance parameter of underground structures to calculate their seismic dynamic response. The relationship between the structural performance parameters and the ground motion index of the peak ground acceleration is established. According to the probabilistic seismic demand analysis method, the vulnerability of underground structures based on performance parameters is analyzed, and the seismic vulnerability curve is drawn to obtain the transcendence probability of the structure's response in the absence of seismic input. As a result, the corresponding damage classes of underground structures with different performance targets are defined.

1. As the peak acceleration increases, the slope of vulnerability curve first increases and then decreases, and the performance level of the structure gradually develops from basically intact to severely damaged. Meanwhile, the probability of damage exceedance increases to varying degrees for each structural level, and the structural vulnerability curve is beginning to levelling off with the severity of structural damage.
2. The failure state probability curve of subway station structure does not monotonically increase along with the ground motion peak acceleration as in the case of the seismic vulnerability curves.
3. The failure probability of subway station structure in failure state under different site conditions when the ground motion intensity reaches small, medium and large earthquake levels.
4. With the increase of soil strength, the vulnerability index of subway station structure under different peak acceleration ground motion decreases correspondingly.

In addition, the subway station structure with established buried depth in saturated soft soil site has certain safety and reliability and can meet the seismic fortification goal of "no damage in small earthquake, repairable in medium earthquake and no collapse in large earthquake".

A large number of historical earthquake data reveals that strong earthquakes are often accompanied by a large number of aftershocks. After the main earthquake, the subway underground structure system in the soft soil site may have entered the nonlinear stage, showing softening plastic deformation, stiffness degradation, and bearing capacity decline. At this time, after the subsequent aftershock sequence, even a small energy release can lead to an exponential increase in seismic risk. Therefore, the potential damage caused by aftershocks with a high probability of secondary energy release cannot be ignored.

## Data Availability

The data used to support the findings of this study are available from the corresponding author upon request.
